# MHD Free Convection and Entropy Generation in a Corrugated Cavity Filled with a Porous Medium Saturated with Nanofluids

**DOI:** 10.3390/e20110846

**Published:** 2018-11-05

**Authors:** Ali J. Chamkha, Fatih Selimefendigil

**Affiliations:** 1 Mechanical Engineering Department, Prince Sultan Endowment for Energy and Environment, Prince Mohammad Bin Fahd University, Al-Khobar 31952, Saudi Arabia; 2 RAK Research and Innovation Center, American University of Ras Al Khaimah, Ras al Khaimah 10021, UAE; 3 Department of Mechanical Engineering, Celal Bayar University, Manisa 45140, Turkey

**Keywords:** MHD free convection, porous medium, nanofluid, second law, finite element method

## Abstract

MHD free convection inside a triangular-wave-shaped corrugated porous cavity with Cu-water nanofluid is numerically studied with the finite element method. The influences of the Grashof number ($10^{4}\leq \mathrm{Gr}\leq 10^{6}$), Hartmann number (0≤Ha≤50), Darcy number ($10^{-4}\leq \mathrm{Da}\;\leq \;10^{-1}$) and solid volume fraction of the nanoparticle (0≤ϕ≤0.05) on the convective flow features are examined. It is observed that increasing the Grashof number and Darcy number enhances the heat transfer, while the effect is opposite for the Hartmann number. As the corrugation frequency of the triangular wave increases, the local and averaged heat transfer rates decrease, which is more effective for higher values of Grashof and Darcy numbers. Normalized total entropy generation increases as the Darcy number and solid volume fraction of the nanoparticles increase and decreases as the Hartmann number increases both for flat and corrugated wall configurations.

## 1. Introduction

Natural convection in porous media has applications in some engineering problems such as in cooling of MEMs, solar collectors, geothermal applications, building insulation materials and many others. Industrial application examples are given in [1,2,3,4]. In some industrial applications such as MEMs, coolers of nuclear reactors and many other magnetic field effects may be relevant [5]. An external magnetic field has the potential to control the convective heat transfer features [6,7,8]. Nanofluid technology with the addition of nano-sized particles to the base fluid was applied in many thermal engineering problems [9,10]. Nanofluids enhance thermal transport [9]. Nanofluids can also be used in applications with magnetic fields, and both thermal conductivity and electrical conductivity of the base fluid are enhanced with the inclusion of the nanoparticles [11,12]. In the numerical study of Mahmoudi et al. [13], free convection in a triangular cavity with nanofluids was examined under the effects of a magnetic field, and the effects of various parameters such as the Rayleigh number, Hartmann number and nanoparticle volume fraction on the convective flow features were analyzed. Ghasemi et al. [14] numerically examined the free convection with a magnetic field for water: ${\mathrm{Al}}_{2}O_{3}$ nanofluid. They noted that depending on the Hartmann and Rayleigh number values, the average Nusselt number may be enhanced or deteriorated. In the literature, there exist several studies of convection in porous media filled with a nanofluid. Sun and Pop [15] examined the natural convection in a porous triangular cavity with nanofluids. They performed the study for different nanofluid types, and they reported that as the solid volume fraction of the nanoparticles increases, the heat transfer in the cavity is enhanced only at low Rayleigh numbers. Bourantas et al. [16] numerically examined the nanofluid natural convection in a porous cavity by using a meshless method, while the Darcy–Brinkman equations were used. The effects of nanofluid in the cooling were examined. Other relevant studies for convective heat transfer in a porous medium saturated by a nanofluid can be found in [17,18,19]. MHD flow in porous enclosures was studied by many researchers [20,21,22,23]. In applications for convection, the geometries can be simplified to some basic shapes such as square, triangular or trapezoidal. Surface corrugations can be used to control the convection in cavities. Hydro-thermal performance in a corrugated channel with nanofluid was numerically examined in [24]. As was shown by Hasan et al. [25], the corrugation amplitude and frequency have a significant impact on the heat transfer rate in natural convection for a deferentially-heated cavity. Hussain et al. [26] numerically examined the natural convection in a tilted sinusoidal corrugated cavity with magnetic field effects.

In convective heat transfer applications, second law analysis can be used to evaluate the system performance [27,28,29,30,31]. A review of the recent developments in entropy generation studies relevant to convective heat transfer is given in [32]. Some application examples of second law analysis with magnetic field and nanofluids can be found in [33,34,35,36,37].

As outlined in the above given literature survey, natural convection in a corrugated enclosure filled with a porous medium saturated with nanofluids under the effects of a magnetic field has many important thermal engineering applications. Some of the mentioned methods (surface corrugation, using nanofluids and an external magnetic field) can also be used as efficient control methods to affect the convective heat transfer features of the thermal engineering design for a porous medium. In this study, second law analysis of the present configuration with surface corrugation was also examined, which adds another novelty to the present study. The results of the present study may be useful in the thermal design of systems, which includes porous media, nanofluid and magnetic field interactions.

## 2. Mathematical Formulation

**Figure 1:**
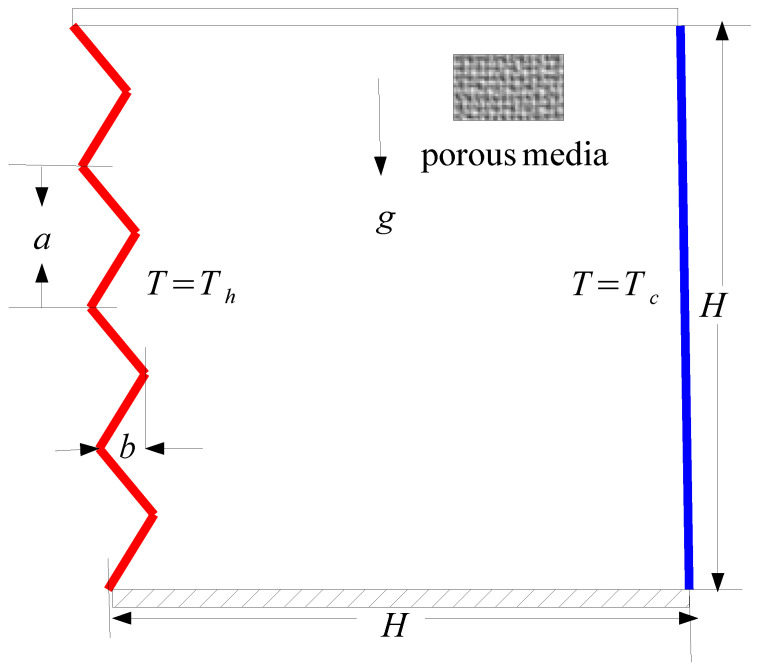
Schematic diagram of the physical model with boundary conditions.

A schematic diagram of the problem for natural convection in a triangular wave-shaped corrugated cavity is shown in Figure 1. The enclosure size is *H*, while the corrugated wave has a length and height as *a* and b=0.2H. The cavity is deferentially heated with isothermal temperatures of $T_{c}$ and $T_{h}$ for the right and left corrugated vertical walls with $T_{h}>T_{c}$. Other walls are adiabatic with no-slip boundary conditions. A uniform vertical magnetic field is imposed. The nanofluid is assumed to saturate the solid matrix, and both are in thermodynamic equilibrium. Thermo-physical properties are shown in Table 1 [13]. Various effects such as joule heating, induced magnetic field and displacement currents are neglected. We assume that the magnetic Reynolds number is much smaller than the induced magnetic field so that it can be neglected when compared to the applied magnetic field.

Conservation equations and entropy generation in a two-dimensional system can be stated as follows [38,39]:

(1) $$\frac{\partial u}{\partial x}+\frac{\partial v}{\partial y}=0,$$

(2) $$u\frac{\partial u}{\partial x}+v\frac{\partial u}{\partial y}=-\frac{1}{\rho_{\mathrm{nf}}}\frac{\partial p}{\partial x}+\nu_{\mathrm{nf}}\left(\frac{\partial^{2}u}{\partial x^{2}}+\frac{\partial^{2}u}{\partial y^{2}}\right)-\frac{\nu_{\mathrm{nf}}}{\kappa }u,$$

(3) $$u\frac{\partial v}{\partial x}+v\frac{\partial v}{\partial y}=-\frac{1}{\rho_{\mathrm{nf}}}\frac{\partial p}{\partial y}+\nu_{\mathrm{nf}}\left(\frac{\partial^{2}v}{\partial x^{2}}+\frac{\partial^{2}v}{\partial y^{2}}\right)-\frac{\nu_{\mathrm{nf}}}{\kappa }v+\beta_{\mathrm{nf}}g\left(T-T_{c}\right)-\frac{\sigma_{\mathrm{nf}}B_{0}^{2}}{\rho_{\mathrm{nf}}}v,$$

(4) $$u\frac{\partial T}{\partial x}+v\frac{\partial T}{\partial y}=\alpha_{\mathrm{nf}}\left(\frac{\partial^{2}T}{\partial x^{2}}+\frac{\partial^{2}T}{\partial y^{2}}\right),$$

(5) $$\begin{array}{c} S=\frac{k_{nf}}{T_{0}^{2}}\left[{\left(\frac{\partial T}{\partial x}\right)}^{2}+{\left(\frac{\partial T}{\partial y}\right)}^{2}\right]+\frac{\mu_{nf}}{T_{0}}\left[2\left({\left(\frac{\partial u}{\partial x}\right)}^{2}+{\left(\frac{\partial v}{\partial y}\right)}^{2}\right)+{\left(\frac{\partial u}{\partial x}+\frac{\partial v}{\partial y}\right)}^{2}\right]\\ +\frac{\mu_{nf}}{T_{0}\kappa }\left(u^{2}+v^{2}\right)+\frac{\sigma_{nf}B_{0}^{2}}{T_{0}}u^{2}. \end{array}$$

In the above representation, entropy generation due to heat transfer, viscous dissipation and magnetic field is shown.

The effective density and specific heat of the nanofluid are defined as:

(6) $$\rho_{nf}=\left(1-\phi \right)\rho_{bf}+\phi \rho_{p},\;\;{\left(\rho c_{p}\right)}_{nf}=\left(1-\phi \right){\left(\rho c_{p}\right)}_{bf}+\phi {\left(\rho c_{p}\right)}_{p}.$$

The thermal expansion coefficient of the nanofluid is given as:

(7) $${\left(\rho \beta \right)}_{nf}=\left(1-\phi \right){\left(\rho \beta \right)}_{bf}+\phi {\left(\rho \beta \right)}_{p}.$$

The Maxwell–Garnett model and Maxwell model were used for the definition of the thermal conductivity and electrical conductivity of the nanofluid as:

(8) $$k_{nf}=k_{f}\left[\frac{\left(k_{p}+2k_{f}\right)-2\phi \left(k_{f}-k_{p}\right)}{\left(k_{p}+2k_{f}\right)+\phi \left(k_{f}-k_{p}\right)}\right],\;\;\;\sigma_{nf}=\sigma_{f}\left[1+\frac{3\left(\frac{\sigma_{p}}{\sigma_{f}}-1\right)\phi }{\left(\frac{\sigma_{p}}{\sigma_{f}}+2\right)-\left(\frac{\sigma_{p}}{\sigma_{f}}-1\right)\phi }\right].$$

The dynamic viscosity of the nanofluid is defined as [40]:

(9) $$\mu_{nf}=\mu_{bf}{\left(1-\phi \right)}^{-0.25}.$$

Neither the viscosity nor the thermal conductivity of the nanofluid as described above include the effects of Brownian motion. Thermophoresis and Brownian motion in nanofluid flow were considered in many studies [41,42,43].

The following dimensionless parameters are used to convert the nondimensional form of equations:

(10) $$\begin{array}{c} \begin{array}{c} X=\frac{x}{H},\;\;Y=\frac{y}{H},\;\;U=\frac{uH}{\alpha },\;\;V=\frac{vH}{\alpha },\;\;P=\frac{p}{\rho_{nf}\alpha^{2}} \end{array},\\ \begin{array}{c} \theta =\frac{T-T_{c}}{T_{h}-T_{c}},\;\;\mathrm{Gr}=\frac{g\beta_{f}\left(T_{h}-T_{c}\right)H^{3}}{\nu_{f}^{2}},\;\;\mathrm{Pr}=\frac{\nu_{f}}{\alpha_{f}} \end{array},\\ \begin{array}{c} \mathrm{Ha}=B_{0}H\sqrt{\frac{\sigma_{nf}}{\rho_{nf}\nu_{f}}},\;\;\mathrm{Da}=\frac{\kappa }{H^{2}} \end{array}. \end{array}$$

Non-dimensional equations are written as follows:

(11) $$\begin{array}{c} \frac{\partial U}{\partial X}+\frac{\partial V}{\partial Y}=0 \end{array},$$

(12) $$\begin{array}{c} U\frac{\partial U}{\partial X}+V\frac{\partial U}{\partial Y}=-\frac{\partial P}{\partial X}+\frac{1}{\mathrm{Pr}}\frac{\nu_{nf}}{\nu_{f}}\left(\frac{\partial^{2}U}{\partial X^{2}}+\frac{\partial^{2}U}{\partial Y^{2}}\right)-\frac{\mathrm{Pr}}{\mathrm{Da}}\frac{\nu_{nf}}{\nu_{f}}U \end{array},$$

(13) $$\begin{array}{c} U\frac{\partial V}{\partial X}+V\frac{\partial V}{\partial Y}=-\frac{\partial P}{\partial Y}+\frac{1}{\mathrm{Pr}}\frac{\nu_{nf}}{\nu_{f}}\left(\frac{\partial^{2}V}{\partial X^{2}}+\frac{\partial^{2}V}{\partial Y^{2}}\right),\\ -\frac{\mathrm{Pr}}{\mathrm{Da}}\frac{\nu_{nf}}{\nu_{f}}V+\frac{\beta_{nf}}{\beta_{f}}{\mathrm{Pr}}^{2}\mathrm{Gr}\theta -{\mathrm{Ha}}^{2}\mathrm{Pr}V, \end{array}$$

(14) $$\begin{array}{c} U\frac{\partial \theta }{\partial X}+V\frac{\partial \theta }{\partial Y}=\frac{\alpha_{nf}}{\alpha_{f}}\left(\frac{\partial^{2}\theta }{\partial X^{2}}+\frac{\partial^{2}\theta }{\partial Y^{2}}\right) \end{array}.$$

Non-dimensional boundary conditions as:

- At the bottom and top walls:
  $U=V=0,\;\frac{\partial \theta }{\partial Y}=0$,
- At the right vertical wall:
  U=V=0,θ=0,
- At the corrugated left wall:
  U=V=0,θ=1.

Local and average Nusselt numbers are calculated as [39]:

(15) $${\mathrm{Nu}}_{s}=-\frac{k_{nf}}{k_{f}}{\left(\frac{\partial \theta }{\partial n}\right)}_{n=0},\;\;{\mathrm{Nu}}_{m}=\int_{0}^{1}{\mathrm{Nu}}_{s}ds.$$

## 3. Solver and Code Validation

**Figure 2:**
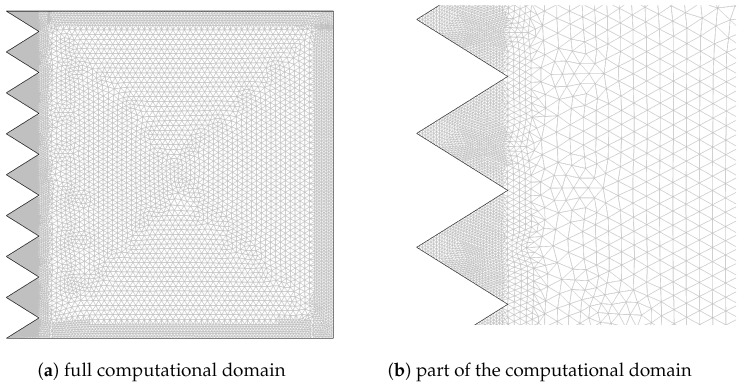
Grid distribution.

**Figure 3:**
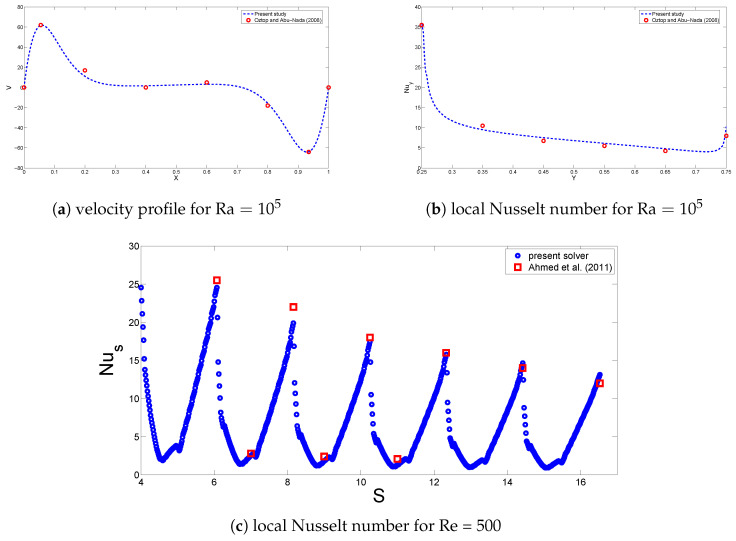
Code verification with the results of Oztop and Abu-Nada [9] (**a**,**b**) and Ahmed et al. [24] (**c**).

The finite element method was used to obtain the solution of governing equations with boundary conditions as described above. The weak form of the governing equations is obtained, and when the approximated flow variables are substituted in the governing equations, residual *R* results, and the weighted average of *R* will be forced to be zero:

(16) $$\int_{\Omega }WRdv=0,$$

where *W* is the weight function, which is chosen from the same set of functions as of the trial functions. A system of nonlinear ordinary differential equations is achieved, which is solved with the Newton–Raphson method. Grid independence tests were performed, and the grid was refined near the walls to resolve the significant gradients in those locations. Table 2 shows the average Nusselt number for various grid sizes $(\mathrm{Gr}=10^{6},\;\mathrm{Ha}=50,\;\phi =0.05,\;\mathrm{Da}=10^{-2}$). Figure 2 demonstrates the grid distribution. The present solver is validated by using the results of Oztop and Abu-Nada [9] for cavity flow at $\mathrm{Ra}=10^{5}$ and the numerical results of Ahmed et al. [24] for corrugated channel flow at Re=500. The results in Figure 3 show sufficient accuracy of the current solver.

## 4. Results and Discussion

**Figure 4:**
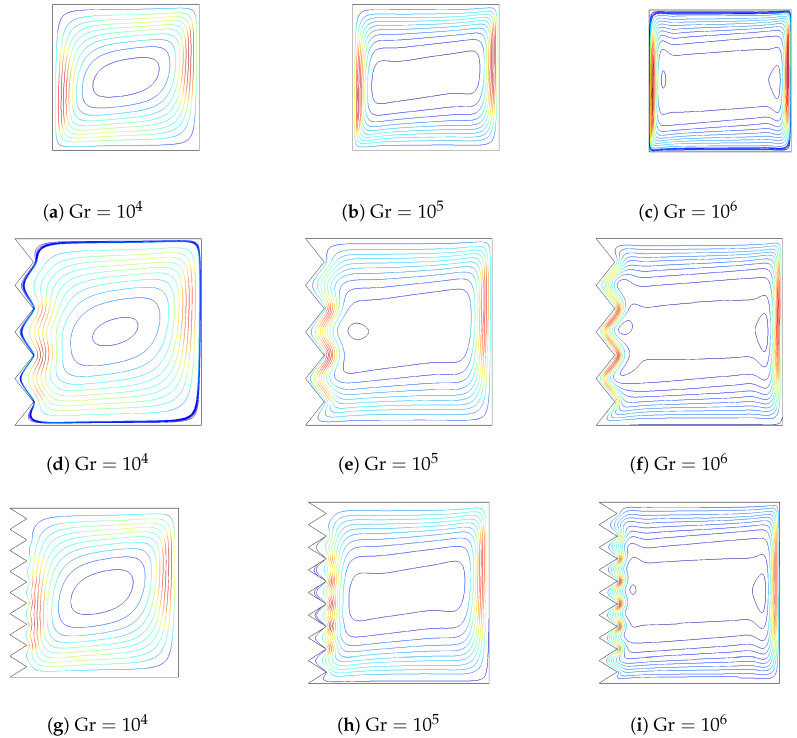
The effect of varying the Grashof number on the streamlines for flat and corrugated walls ($\mathrm{Ha}=20,\;\mathrm{Da}=10^{-2},\;\phi =0.02$).

**Figure 5:**
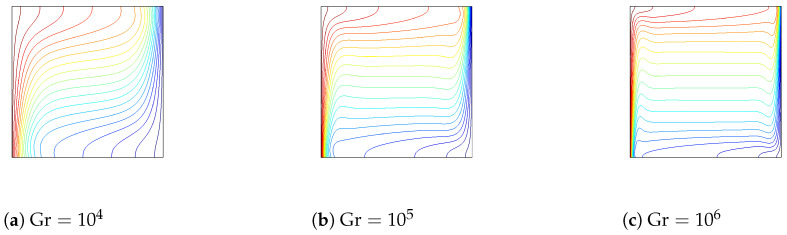

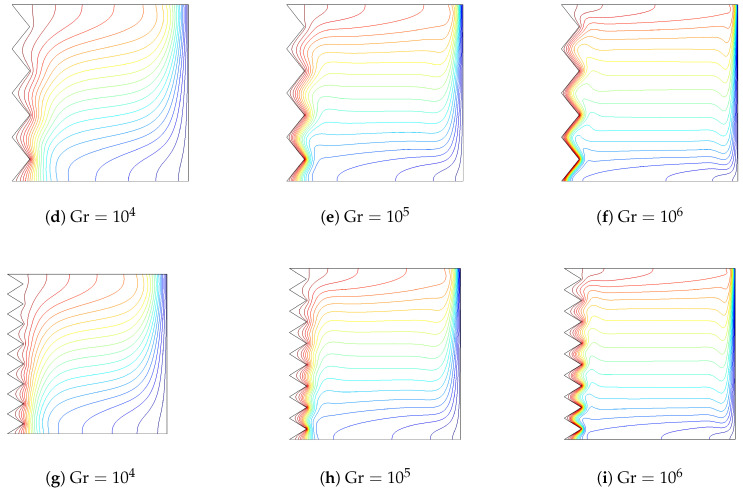
The effect of varying the Grashof number on the isotherms for flat and corrugated walls ($\mathrm{Ha}=20,\;\mathrm{Da}=10^{-2},\;\phi =0.02$).

**Figure 6:**
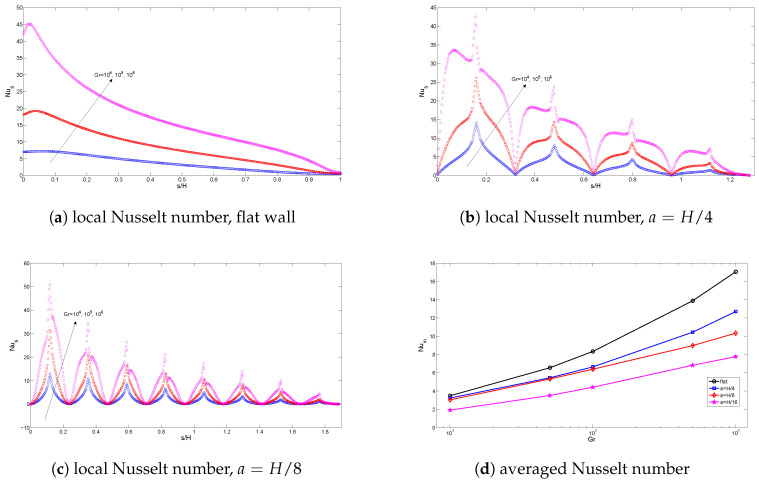
Local and averaged Nusselt number along the flat and corrugated walls for various Grashof numbers ($\mathrm{Ha}=20,\;\mathrm{Da}=10^{-2},\;\phi =0.02$).

The effects of varying the Grashof number on the variation of flow and thermal patterns are shown in Figure 4 and Figure 5 ($\mathrm{Ha}=20,\;\mathrm{Da}=10^{-2},\;\phi =0.02$). A single main recirculation cell is observed for all values of Grashof numbers. When the Grashof number value is augmented, the flow strength increases. At a low Grashof number, the isotherms are parallel to the vertical walls, indicating the characteristics of the conduction-dominated regime. By increasing the Grashof number, the flow strength increases, and temperature gradients near the hot and cold wall become significant. The effects of the corrugated wall on the streamlines and isotherms are also shown in Figure 4 and Figure 5. The global features of the flow and thermal patterns are not disturbed, except in the vicinity of the left wall. The streamlines and isotherms mimic the wall’s profile. The value of the maximum stream function increases with surface corrugation. The variation of the local and averaged Nusselt numbers for various Grashof numbers is shown in Figure 6 for flat and corrugated walls. As the Grashof number increases, heat transfer is locally enhanced along the heated wall for all configurations. The effect of the frequency of the triangular wave of the corrugated surface is also demonstrated in Figure 6. As is seen from the figure, the imposed frequency of the triangular wave affects the variation of the local heat transfer for the corrugated wall. As the frequency of the triangular wave increases, averaged heat transfer increases, and this effect is significant at higher Grashof numbers. This is due to the variation of the thermal and flow patterns adjacent to the boundary with respect to the change in the frequency. The increase in frequency reduces the space for the circulation adjacent to the boundary of the heated wall. This result is supported by Hasan et al. [25].

**Figure 7:**
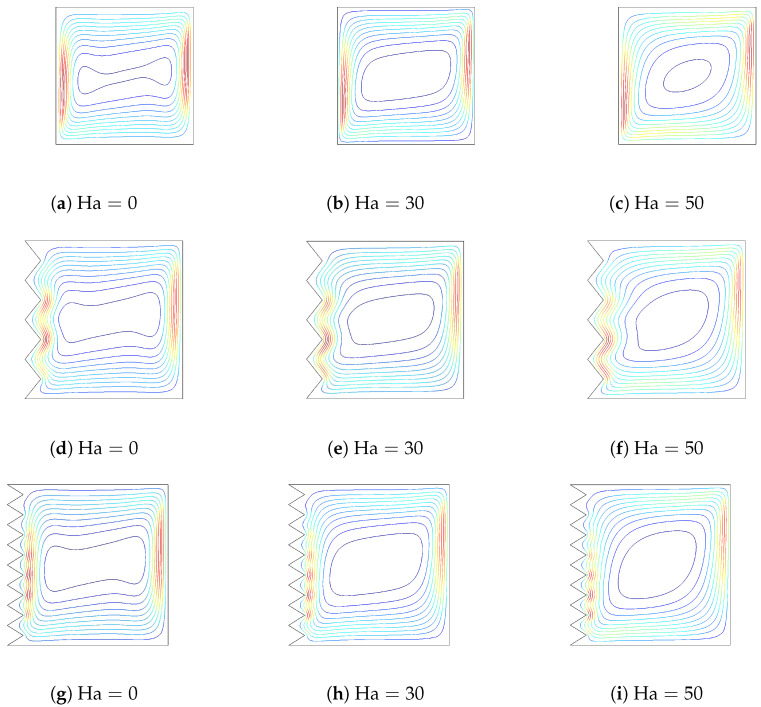
The effect of varying the Hartmann number on the streamlines for flat and corrugated walls ($\mathrm{Gr}=5\times 10^{4},\;\mathrm{Da}=10^{-2},\;\phi =0.02$).

**Figure 8:**
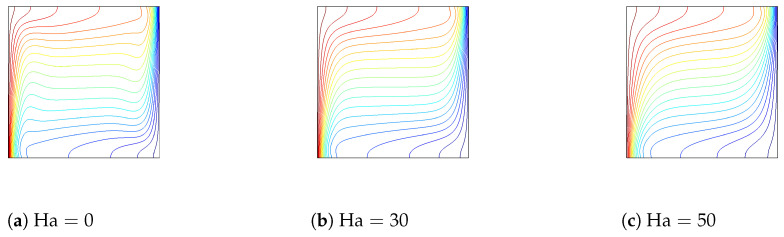

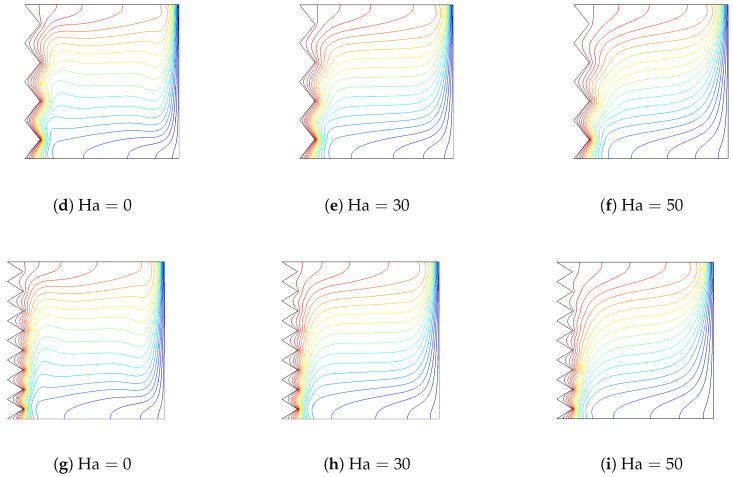
The effect of varying the Hartmann number on the isotherms for flat and corrugated walls ($\mathrm{Gr}=5\times 10^{4},\;\mathrm{Da}=10^{-2},\;\phi =0.02$).

**Figure 9:**
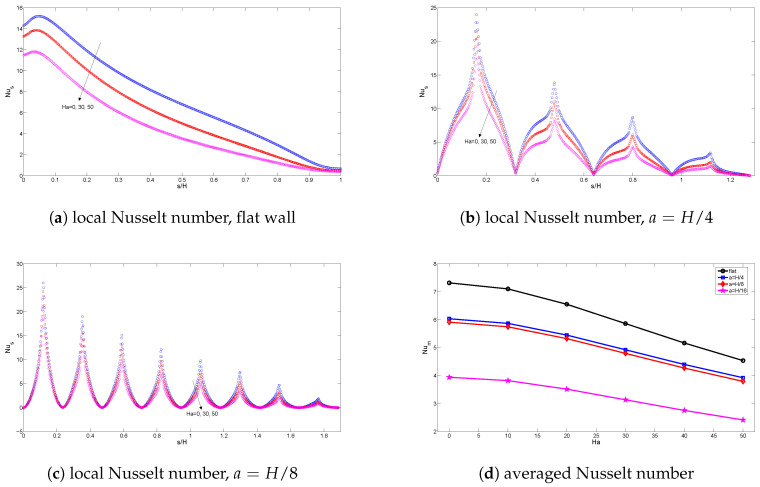
Local and averaged Nusselt number along the flat and corrugated wall for various Hartmann numbers ($\mathrm{Gr}=5\times 10^{4},\;\mathrm{Da}=10^{-2},\;\phi =0.02$).

The influences of varying the Hartmann number on streamlines and isotherms ($\mathrm{Gr}=5\;\times \;10^{4}$, $\mathrm{Da}=10^{-2},$ ϕ=0.02) are demonstrated in Figure 7 and Figure 8. The strength of convection decreases as the Hartmann number increases for flat and corrugated wall configurations. When the value of the Hartmann number rises, a less dense arrangement of isotherms is obtained along the corrugated wall. The influence of the Hartmann number on the variation of the local and average Nusselt number is shown in Figure 9. Nusselt numbers decrease as the value of the Hartman number rises, which is due to the convective heat transfer suppression with a magnetic field, both for a flat wall and a corrugated wall configuration. These results are supported by [13,14].

**Figure 10:**
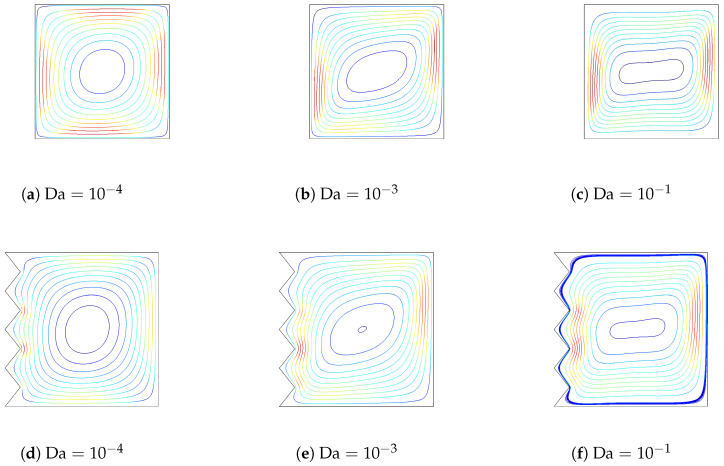

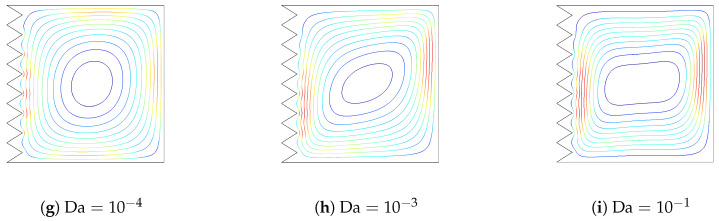
The effect of varying the Darcy number on the streamlines for flat and corrugated walls ($\mathrm{Gr}=10^{4},\;\mathrm{Ha}=10,\;\phi =0.02$).

**Figure 11:**
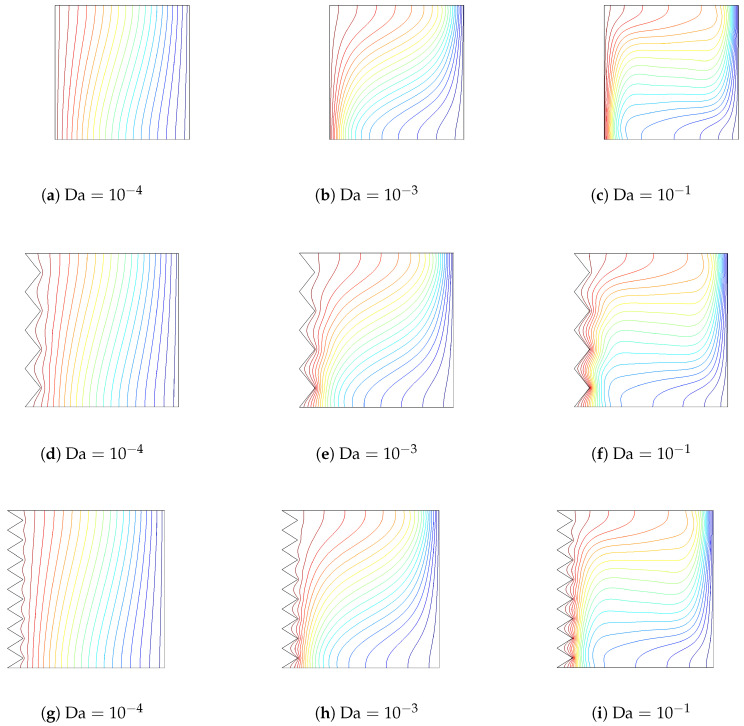
The effect of varying the Darcy number on the isotherms for flat and corrugated walls ($\mathrm{Gr}=10^{4},\;\mathrm{Ha}=10,\;\phi =0.02$).

**Figure 12:**
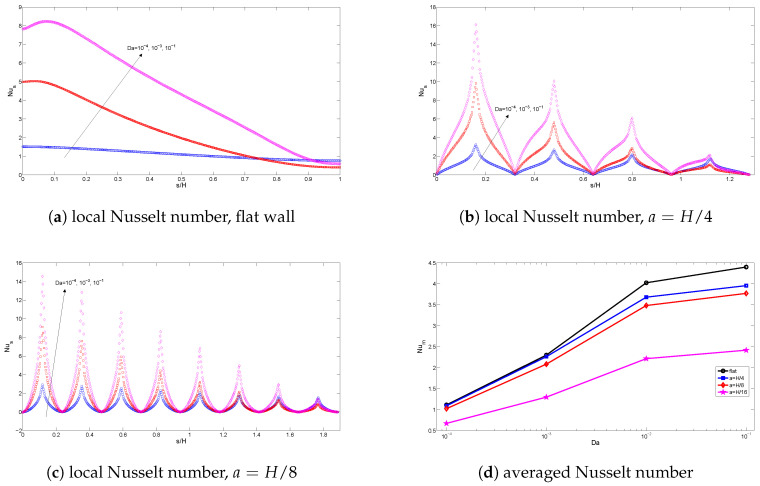
Local and averaged Nusselt number along the flat and corrugated walls for various Darcy numbers ($\mathrm{Gr}=10^{4},\;\mathrm{Ha}=10,\;\phi =0.02$).

**Figure 13:**
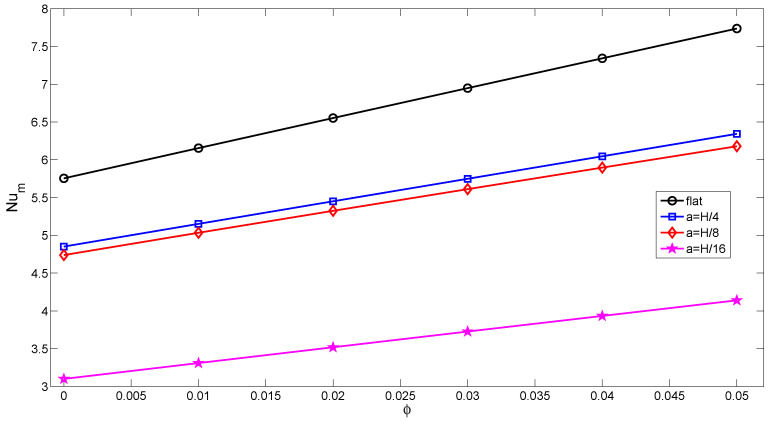
Averaged Nusselt number along the flat and corrugated walls for various nanoparticle volume fractions ($\mathrm{Gr}=5\times 10^{4},\;\mathrm{Ha}=20,\;\mathrm{Da}=10^{-2}$).

**Figure 14:**
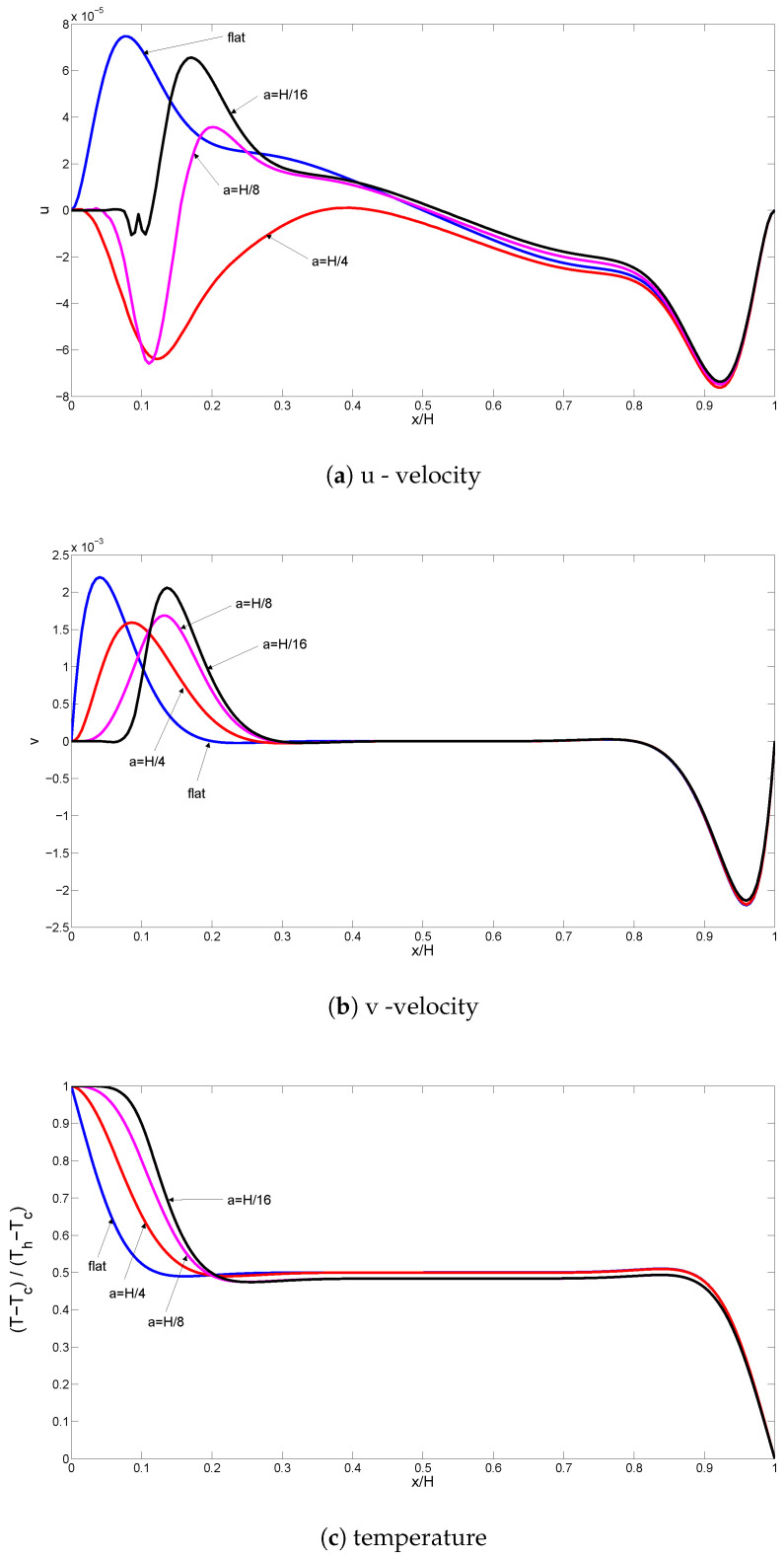
Velocity and temperature profiles along the horizontal plane at y=0.5H for flat and corrugated walls ($\mathrm{Gr}=10^{5},\;\mathrm{Ha}=20,\;\mathrm{Da}=10^{-2},\;\phi =0.02$).

Figure 10 and Figure 11 demonstrate the effects of varying the Darcy number on streamlines and isotherms for the flat wall and corrugated wall cases ($\mathrm{Gr}=10^{4},\;\mathrm{Ha}=10,\;\phi =0.02$). For low values of the Darcy number, fluid motion is not signification, and this can contribute to the high resistance of the medium. Convection becomes significant for higher values of the Darcy number with flat and corrugated wall configurations of the cavity. Conduction is dominant for low values of the Darcy number, which is demonstrated in Figure 11, while the isotherms are distributed approximately parallel to the vertical walls. The main cell is elongated horizontally as the Da number increases, as shown in Figure 10. An increment in Da results in increased temperature gradients near the heated wall, which can contribute to the enhanced permeability of the medium. In this case, the resistance from the boundary friction has been reduced with increasing Da number. Local and averaged heat transfer distributions for various Da numbers are shown in Figure 12. Local heat transfer is augmented for higher values of the Darcy number when both flat and corrugated wall configurations are considered. The effect of Da number on the average Nusselt number is less for the flat wall and corrugated wall with a=H/4 and a=H/8 for a low Da number due to the dominance of conduction. As the Da number increases, the effect of corrugation on the averaged heat transfer is more pronounced. The effect of nanofluid on the averaged heat transfer is demonstrated in Figure 13 ($\mathrm{Gr}=5\times 10^{4},\;\mathrm{Ha}=20,\;\mathrm{Da}=10^{-2}$). Heat transfer enhancements of 34.4%, 30.7%, 30.4% and 33.5% are achieved for the nanofluid with a volume fraction of ϕ=0.05 when it is compared to the base fluid (ϕ=0) for the flat wall and corrugated walls with a=H/4, a=H/8 and a=H/16, respectively. The distribution of velocity and temperature along a horizontal plane located at y=0.5H are shown in Figure 14 ($\mathrm{Gr}=10^{5},\;\mathrm{Ha}=20,\;\mathrm{Da}=10^{-2},\;\phi =0.02$) for flat and corrugated wall configurations. These profiles are affected from the corrugation frequency in the vicinity of the heated wall. Temperature increases with wall corrugation and its frequency. A shift and reduction in the peak values of the velocity components are seen with wall corrugation.

**Figure 15:**
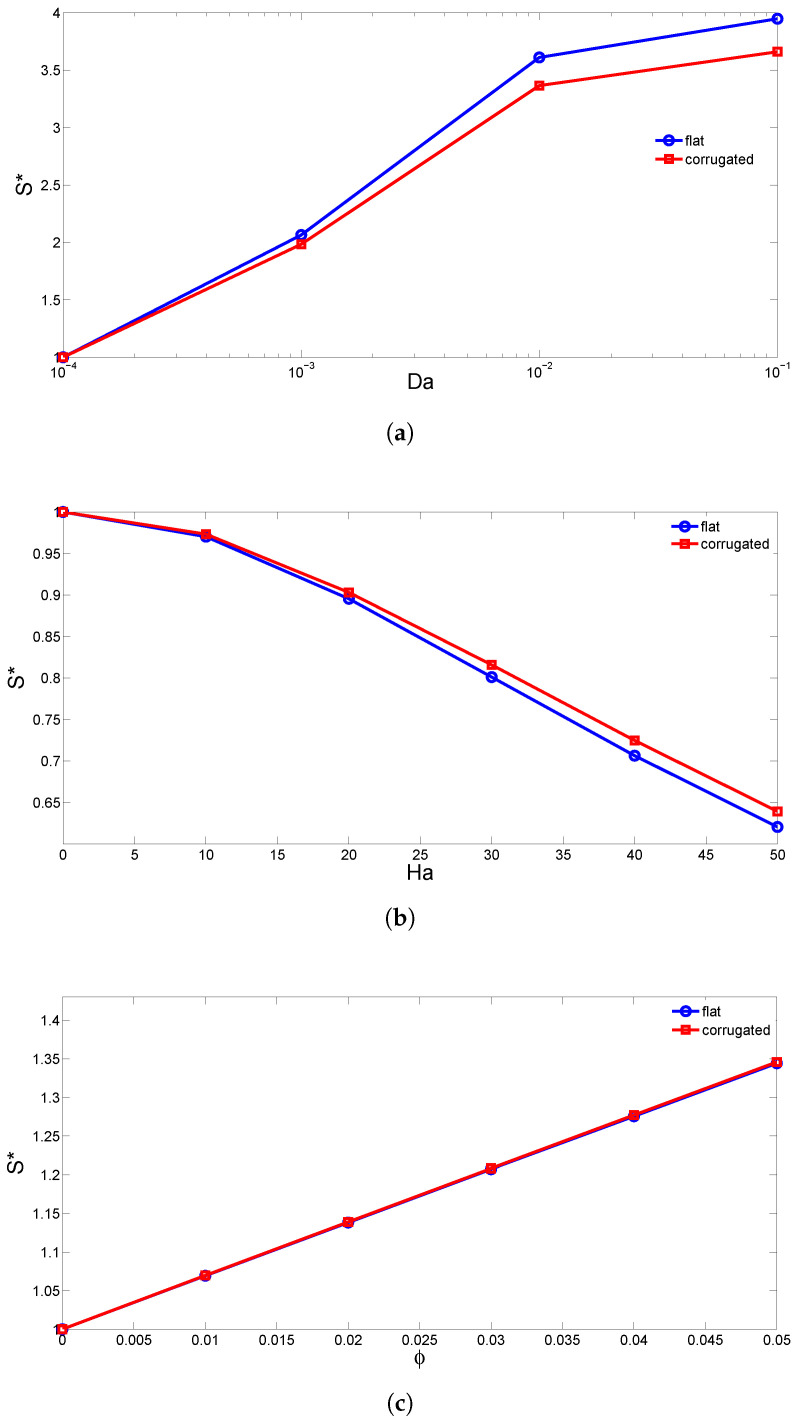
The effects of varying the Darcy number (**a**) Hartmann number (**b**) nanoparticle volume fraction (**c**) on the normalized total entropy generation within the cavity.

Normalized total entropy generation (normalized by the total entropy generation value for the first parameter) is shown in Figure 15 for various Darcy numbers, Hartmann numbers and nanoparticle volume fractions for both a flat wall and a corrugated wall with the a=H/4 case. The total entropy generation increases as the value of the Darcy number increases due to the fluid friction irreversibility. When the value of the Hartmann number rises, the total entropy generation deteriorates, which is due to the suppression of the convection with the magnetic field strength. An increment in the solid volume fraction of nanoparticles resulted in the enhancement of the total entropy generation rate, which is due to the rise of heat transfer and fluid friction irreversibility. The discrepancy between the normalized entropy generation of the flat and corrugated wall cases increases as the values of the Darcy and Hartmann numbers rise.

## 5. Conclusions

A numerical study of MHD free convection in a triangular wave-shaped corrugated cavity filled with a porous medium saturated with nanofluids was analyzed. The following conclusions can be stated as:

- Increasing the Grashof number and Darcy number results in heat transfer enhancement while keeping other parameters fixed.
- The imposed frequency of the triangular wave affects the variation of the local Nusselt number for the corrugated wall. The velocity and temperature distributions are very sensitive to the corrugation frequency near the heated wall.
- Increasing the corrugation frequency reduces the local and average Nusselt number.
- The effect of the frequency of the triangular wave on the average Nusselt number is significant at higher values of the Grashof and Darcy numbers.
- The magnetic field reduced the convective heat transfer.
- Thirty-four-point-four percent of the average Nusselt number enhancement is obtained for the nanofluid with a nanoparticle volume fraction of ϕ=0.05 when it is compared to the base fluid (ϕ=0) for the flat wall.
- The normalized total entropy generation increases as the values of the Da number (due to the fluid friction irreversibility) and ϕ increase and the Ha number decreases (suppression of convection). The discrepancy between the normalized entropy generation for a flat and a corrugated wall is more pronounced for the highest values of the Darcy and Hartmann numbers.

An extension of this study may be considered to include the influence of corrugation amplitude, various corrugation types (rectangular, trapezoidal, sinusoidal, etc.), different nanofluid types and transient flow effects.

## Figures and Tables

**Table 1:** Thermophysical properties.

Property	Water	Cu
ρ	997.1	8954
$\;c_{p}$	4179	383
k	0.6	400
β	$2.1\times 10^{-4}$	$1.67\times 10^{-5}$
σ	0.05	$5.97\times 10^{7}$

**Table 2:** Grid independence test.

Grid Name	Number of Elements	Averaged Nusselt Number
G1	1136	9.232
G2	2796	9.983
G3	4544	10.483
G4	18,176	11.087
G5	52,624	11.184

## Abbreviations/Nomenclature

a	length of triangular wave (m)
b	height of triangular wave (m)
**B${}_{0}$**	magnetic field strength
**Da**	Darcy number, $\frac{\kappa }{H^{2}}$
**Gr**	Grashof number, $\frac{g\beta_{f}(T_{h}-T_{c}H^{3}}{\nu_{f}^{2}}$
*h*	local heat transfer coefficient (W/m${}^{2}$K)
**Ha**	Hartmann number, $B_{0}H\sqrt{\frac{\sigma_{nf}}{\rho_{nf}\nu_{f}}}$
*k*	thermal conductivity (W/m·K)
*H*	length of the enclosure (m)
*n*	unit normal vector
Nu	local Nusselt number
*p*	pressure (Pa)
*P*	non-dimensional pressure
Pr	Prandtl number, $\frac{\nu_{f}}{\alpha_{f}}$
*T*	temperature (K)
*u, v*	x-y velocity components (m/s)
*U, V*	dimensionless velocity components
*x, y*	Cartesian coordinates (m)
*X, Y*	dimensionless coordinates
Greek characters:	
α	thermal diffusivity (m${}^{2}$/s)
β	expansion coefficient (1/K)
ϕ	nanoparticle volume fraction
θ	non-dimensional temperature,
$\frac{T-T_{c}}{T_{h}-T_{c}}$	
ν	kinematic viscosity (m${}^{2}$/s)
ρ	density of the fluid (kg/m${}^{3}$)
σ	electrical conductivity (S/m)
Subscripts:	
*c*	cold wall
*m*	average
*h*	hot wall
